# Sustained monomorphic ventricular tachycardia as the first manifestation of probable cardiac sarcoidosis: A case report

**DOI:** 10.1016/j.radcr.2026.06.170

**Published:** 2026-07-25

**Authors:** Muhammad Sanusi, Fadi Haddad, Fawaz Mohammed, Parjanya Bhatt, Dinakaran Umashankar, Faiza Zakaria, Sai Mrudulla Alla, Abdirahman Ahmed, Yash Varma, Christian Toquiqa Gahona, Stacy Loree, Aaref Badshah, Abdul Halabi, Rajiv Nair

**Affiliations:** a Department of Medicine, Trinity Health Oakland, Wayne State University, Pontiac, MI 48341, USA; b Department of Cardiology, Trinity Health Oakland, Wayne State University, Pontiac, MI 48341, USA; c Authority Health, Michigan State University College of Osteopathic Medicine, Detroit, MI 48202, USA

**Keywords:** Ventricular tachycardia, Cardiac sarcoidosis, Cardiac magnetic resonance, Echocardiography, Positron emission tomography

## Abstract

Cardiac sarcoidosis is an inflammatory heart disease that can cause conduction block, ventricular arrhythmias, and cardiomyopathy. Sustained monomorphic ventricular tachycardia is a high-risk presentation and may be the first sign of disease. A 69-year-old man with paroxysmal atrial fibrillation and chronic diastolic heart failure presented with palpitations, dizziness, and shortness of breath after a physical-therapy session. In the emergency department, he was found to be in atrial fibrillation with rapid ventricular response and frequent sustained runs of monomorphic ventricular tachycardia at rates >200 beats/min. Intravenous amiodarone was started. Echocardiography showed mildly reduced left ventricular ejection fraction (46%) and apical hypokinesis. Coronary angiography revealed only minimal nonobstructive coronary artery disease. Cardiac magnetic resonance imaging demonstrated mildly reduced left ventricular function (45%) and a nonischemic pattern of late gadolinium enhancement in the basal anteroseptal, basal inferoseptal, mid-inferoseptal, and inferior segments, highly suggestive of cardiac sarcoidosis. Endomyocardial biopsy was not pursued because of its low sensitivity and invasive risk, and the patient met contemporary criteria for “probable” cardiac sarcoidosis based on imaging and clinical findings. A targeted evaluation for extracardiac disease and infiltrative mimics was unremarkable. Further testing, including positron emission tomography, was planned as outpatient testing. He underwent implantation of a single-chamber implantable cardioverter–defibrillator for secondary prevention and was discharged in stable condition. This case highlights cardiac sarcoidosis as an important cause of sustained ventricular tachycardia in patients with non-ischemic scar on cardiac magnetic resonance and minimal coronary disease. Modern guidelines allow a working diagnosis without biopsy, relying instead on cardiac magnetic resonance and positron emission tomography.

## Introduction

Sarcoidosis is a systemic granulomatous disease that can affect multiple organs, including the heart. Cardiac involvement is often clinically silent but is an important cause of conduction disease, ventricular arrhythmias, and heart failure, and it contributes substantially to sudden cardiac death in this population [1,2].

Historically, endomyocardial biopsy was considered the diagnostic gold standard for cardiac sarcoidosis. However, biopsy has limited sensitivity because myocardial granulomatous infiltration is patchy, and sampling often misses affected areas; it also carries procedural risks, such as perforation and arrhythmia [1,3]. Contemporary expert consensus statements from the Heart Rhythm Society (HRS) and the Japanese Circulation Society, as well as a recent American Heart Association (AHA) scientific statement, recognize these limitations and now permit a diagnosis of “probable” cardiac sarcoidosis based on characteristic clinical features and imaging findings without requiring myocardial tissue in all patients [[3], [4], [5]].

Cardiac magnetic resonance imaging with late gadolinium enhancement (LGE) and 18F-fluorodeoxyglucose positron emission tomography (FDG-PET) have become central to this non-invasive diagnostic strategy. Cardiac magnetic resonance (CMR) identifies nonischemic scar patterns strongly associated with sarcoid involvement and adverse outcomes, while PET detects active inflammation and helps guide immunosuppressive therapy [1,2,6].

Patients with sustained ventricular tachycardia (VT) and structural heart disease are at high risk for recurrent arrhythmias and sudden cardiac death. The 2017 AHA/ACC/HRS guideline recommends ICD implantation for secondary prevention in such patients, and the HRS cardiac sarcoidosis consensus statement specifically endorses ICD therapy for sarcoid-related VT [5,7].

The following case describes a patient who presented with sustained monomorphic VT, minimal coronary artery disease, and a CMR pattern strongly suggestive of cardiac sarcoidosis. The diagnosis and treatment strategy relied on advanced imaging without endomyocardial biopsy and followed current guideline recommendations.

## Case presentation

The patient was a 69-year-old man who came to the emergency department because his heart had been racing for several hours. His medical history included paroxysmal atrial fibrillation treated with apixaban, first-degree atrioventricular block, chronic diastolic heart failure, hypertension, hyperlipidemia, chronic obstructive pulmonary disease, and long-standing tobacco use.

On the evening of the presentation, He had attended a routine physical-therapy session for a hand injury. Shortly after returning home, He developed a sudden sensation that his heart was pounding rapidly and irregularly. He was familiar with brief episodes of atrial fibrillation and usually felt palpitations that resolved within minutes; however, this episode felt different. The palpitations were accompanied by dizziness, a sense of lightheadedness, mild shortness of breath, and a vague pressure in his chest. He did not experience frank chest pain, near-syncope, or loss of consciousness. When the symptoms persisted for more than 2 hours, he decided to seek medical attention.

In the emergency department, he appeared uncomfortable but was alert and oriented. His blood pressure was 118/76 mmHg, his heart rate was fluctuating, and at times extremely rapid, respiratory rate was 20 breaths per minute, and oxygen saturation was 96% on room air. On cardiovascular examination, the rhythm was markedly irregular and tachycardic, without murmurs, rubs, or gallops. His lungs were clear, there was no jugular venous distension, and no lower-extremity edema was noted.

A 12-lead electrocardiogram revealed atrial fibrillation with rapid ventricular response. During monitoring, the rhythm repeatedly transitioned into wide-complex monomorphic VT at rates exceeding 200 beats per minute. Each episode lasted long enough to qualify as sustained VT, but he remained awake and maintained blood pressure. Intravenous amiodarone was administered as a bolus followed by continuous infusion, and external defibrillator pads were applied. He was admitted to the cardiac care unit for close observation.

Overnight, continuous telemetry showed frequent non-sustained runs of VT despite ongoing amiodarone therapy, although he continued to be hemodynamically stable. The following day, transthoracic echocardiography demonstrated mildly reduced left ventricular systolic function with an ejection fraction of 46%, mild concentric left ventricular hypertrophy, and apical hypokinesis with a possible apical thrombus. Right ventricular size was normal with mildly increased wall thickness and preserved systolic function. Because an apical thrombus could not be excluded, he was started on intravenous heparin while further diagnostics were arranged.

Coronary angiography was performed to evaluate for an ischemic cause of his arrhythmia. The study showed a right-dominant coronary circulation with a normal left main, a 20% proximal lesion in the left anterior descending artery with normal distal flow, a normal left circumflex artery, and a normal right coronary artery. There were no obstructive coronary artery disease and no culprit lesion to explain scar-related VT.

To better characterize his cardiomyopathy and evaluate for infiltrative or inflammatory disease, a cardiac magnetic resonance study was obtained. CMR showed a mildly dilated left ventricle with an ejection fraction of 45% and mild thickening of the basal septum. Regional wall motion analysis revealed mild hypokinesis of the basal anteroseptal, basal inferoseptal, and apical segments. Importantly, late gadolinium enhancement demonstrated a patchy, mid-myocardial to subepicardial pattern involving the basal anteroseptal, basal inferoseptal, mid-inferoseptal, and inferior segments. No definite left ventricular thrombus was identified, and the right ventricle was mildly dilated but functionally preserved with an ejection fraction of 57% (Fig. 1, Fig. 2, Fig. 3, Fig. 4).

**Fig. 1:**
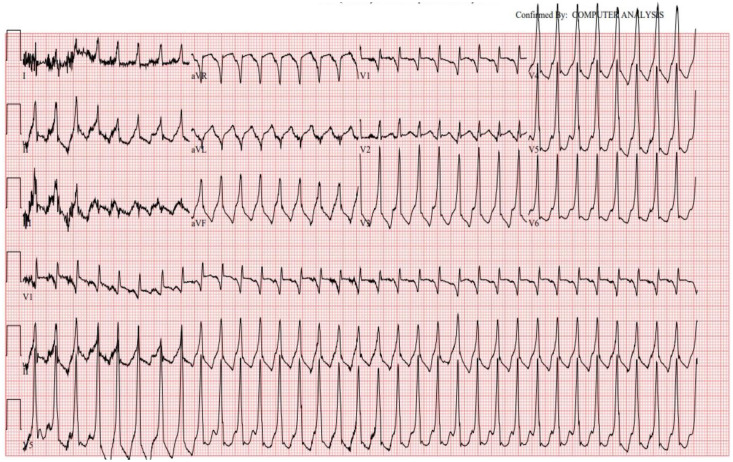
Electrocardiogram showing wide complex tachycardia with a ventricular rate of 202 beats per minute.

**Fig. 2:**
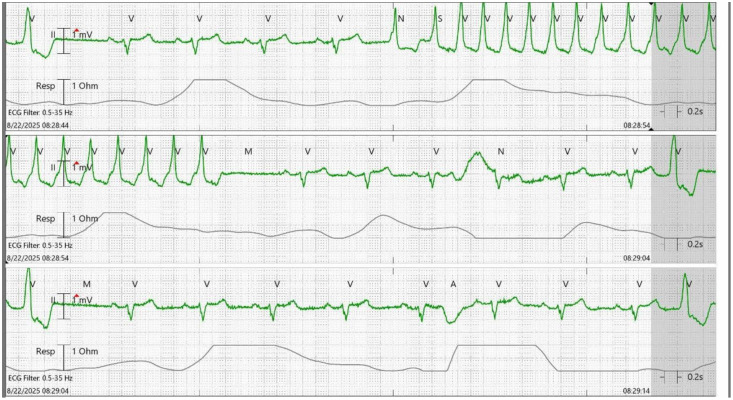
Telemetry strip showing wide complex tachycardia.

**Fig. 3:**
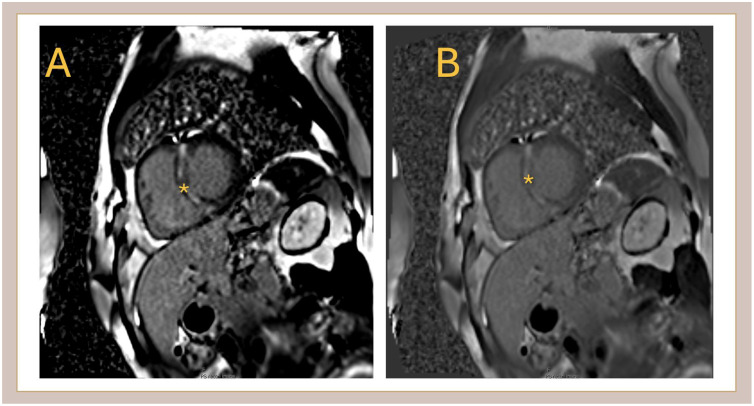
Cardiac MRI late gadolinium enhancement (LGE) showing (A) magnitude-reconstructed LGE image short-axis view demonstrating significant hyperenhancement within the ventricular septum (asterisk). (B) Phase-sensitive inversion recovery (PSIR) image showing healthy muscle (appearing dark gray) better defining the borders of septal scar (asterisk).

**Fig. 4:**
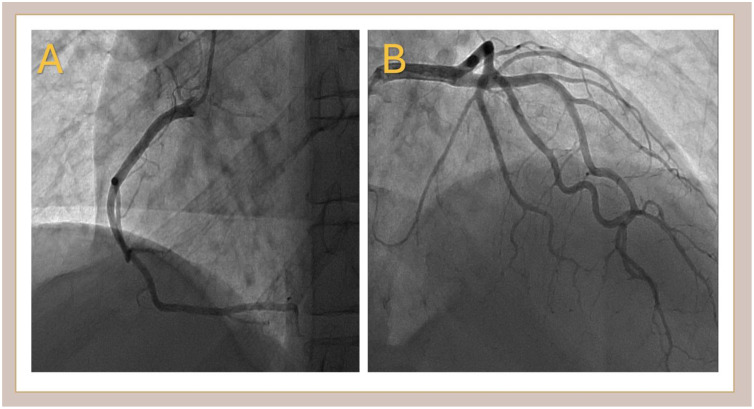
(A) Coronary angiogram of the right coronary artery showing minimal disease in the right coronary artery. (B) Left coronary system angiography demonstrating minimal disease in the left anterior descending and left circumflex coronary artery.

This LGE pattern was highly suggestive of cardiac sarcoidosis rather than ischemic heart disease or amyloidosis. Based on these findings, the suspected apical thrombus was ruled out, and intravenous heparin was discontinued. After multidisciplinary discussion, endomyocardial biopsy was not pursued because of its low diagnostic yield and invasive risk, and because the combination of clinical presentation, exclusion of coronary disease, and characteristic CMR findings met established criteria for probable cardiac sarcoidosis.

A serum ACE level was not obtained during the index admission. Laboratory evaluation at that time showed a mild high-sensitivity troponin rise with plateau (19, 34, 58, 60, 55, and 55 ng/L), normal calcium (8.8 mg/dL), normal alkaline phosphatase (91 U/L), normal AST and ALT (22 and 20 U/L), normal creatinine (0.84 mg/dL), normal white blood cell count (7.5 × 10^9^/L), normal hemoglobin (14.5 g/dL), and normal 25-hydroxy vitamin D level (37.2 ng/mL). These findings did not show clear biochemical evidence of systemic sarcoid-related organ involvement during hospitalization. Of note, serum ACE has limited sensitivity and specificity for cardiac sarcoidosis and is not included in the current Heart Rhythm Society, Japanese Circulation Society, or American Heart Association diagnostic criteria; therefore, its absence does not alter the imaging-based classification of probable cardiac sarcoidosis (Table 1).

**Table 1:** Summary of investigations performed to exclude infiltrative/inflammatory mimics and to screen for extracardiac sarcoidosis.

Investigation	Result/interpretation
High-sensitivity troponin (serial)	Rise-and-fall pattern, peak ∼60 ng/L—myocardial injury attributable to sustained ventricular tachycardia
Serum calcium	8.8 mg/dL (normal)
25-Hydroxy-vitamin D	37.2 ng/mL (normal)
High-sensitivity CRP	1.3 mg/L
NT-proBNP	412 pg/mL (mildly elevated)
QuantiFERON-TB Gold	Negative
*Borrelia* (Lyme) serology	Negative
Fungal serology panel	Negative
Serum protein electrophoresis	Negative
Serum immunofixation	Small IgA-lambda M-protein, too small to quantify
Serum free light chains	Kappa 2.96, lambda 3.24 mg/dL; ratio 0.91 (normal)
Urine protein electrophoresis	Trace albumin; no monoclonal protein
Cardiac FDG-PET	Pending
Tc-99m pyrophosphate (PYP) scan	Pending

Given his sustained monomorphic VT in the setting of structural heart disease and probable cardiac sarcoidosis, he met guideline-based indications for an ICD for secondary prevention of sudden cardiac death. On hospital day 6, he underwent successful implantation of a Biotronik DX single-chamber ICD without complications. He remained free of further sustained VT, continued amiodarone with a plan for gradual tapering, and was discharged home in stable condition with follow-up arranged in cardiology and electrophysiology clinics.

Following discharge, additional outpatient evaluation was arranged to assess for extracardiac sarcoidosis and alternative infiltrative cardiomyopathies. Recent testing showed high-sensitivity CRP of 1.3 mg/L and NT-proBNP of 412 pg/mL. Lyme disease screening and QuantiFERON-TB Gold were negative. Serum protein electrophoresis was negative, urine protein electrophoresis showed a small amount of albumin, and serum free light-chain testing showed a normal kappa/lambda ratio. Immunofixation demonstrated a very small IgA lambda M-protein that was too small to quantify. A cardiac FDG-PET scan was ordered to evaluate for active myocardial inflammation and extracardiac FDG-avid disease. A nuclear PYP scan and myocardial perfusion study were also ordered to evaluate alternative diagnoses, including amyloidosis further. Therefore, the case is best classified as probable cardiac sarcoidosis, with isolated cardiac sarcoidosis remaining a possibility pending completion of outpatient testing.

## Discussion

This case demonstrates how sustained monomorphic VT in a patient with only mild non-obstructive coronary disease and a nonischemic LGE pattern on CMR can reveal underlying cardiac sarcoidosis. The diagnostic and therapeutic approach followed the evolution of current guidelines, which increasingly favor advanced imaging and reserve biopsy for selected situations.

### Cardiac sarcoidosis and ventricular arrhythmias

Cardiac sarcoidosis results from non-caseating granulomatous inflammation that can infiltrate the myocardium, conduction system, and coronary microvasculature [1,2]. Over time, inflammation leads to scarring, electrical heterogeneity, and re-entry circuits that predispose to ventricular arrhythmias and sudden cardiac death [1,7]. Ventricular arrhythmias are a leading cause of death in patients with cardiac sarcoidosis and may be the first clinical manifestation of the disease [1,7]. In our patient, the combination of pre-existing conduction disease (first-degree AV block), mild nonischemic cardiomyopathy, and new sustained monomorphic VT was highly compatible with this pathophysiology.

### Limitations of endomyocardial biopsy

Although histologic demonstration of noncaseating granulomas remains the definitive proof of cardiac sarcoidosis, endomyocardial biopsy has important limitations. Granulomatous lesions are often patchy and localized, making them easy to miss with standard right-ventricular septal sampling, resulting in low sensitivity, often quoted at around 20%-30% [1]. Biopsy also carries risks, including perforation, tamponade, and procedure-induced arrhythmias [1].

Recognizing these limitations, the 2014 HRS expert consensus statement introduced diagnostic pathways that allow a clinical diagnosis of cardiac sarcoidosis without myocardial biopsy when extracardiac sarcoidosis is present or when cardiac imaging shows typical abnormalities in the appropriate clinical context [3]. The Japanese Circulation Society guideline and the 2024 AHA scientific statement further support this approach, emphasizing non-invasive criteria that incorporate imaging findings from CMR and FDG-PET [4,6]. For many patients, including the one described here, a biopsy would likely not change management but would expose them to additional risk.

The 2024 European clinical consensus statement similarly endorses a multimodality, imaging-led diagnostic pathway and emphasizes a thorough search for extracardiac involvement, both to support the diagnosis and because the presence and distribution of disease influence immunosuppressive and device management [8]. In keeping with this, our patient underwent biochemical and serological screening together with planned FDG-PET; when extracardiac disease cannot be demonstrated, the working diagnosis is best framed as probable isolated cardiac sarcoidosis, which remains a recognized but underdiagnosed entity and one in which histological confirmation is especially difficult to obtain.

### Central role of CMR and FDG-PET

CMR with LGE has transformed the evaluation of suspected cardiac sarcoidosis. In a landmark study, Patel et al. [9] showed that contrast-enhanced CMR was more than twice as sensitive as older clinical criteria for detecting cardiac involvement and that the presence of myocardial damage on CMR was associated with worse outcomes [9]. Subsequent work by Greulich et al and others demonstrated that LGE in non-ischemic, patchy mid-myocardial or sub-epicardial patterns, particularly involving the basal septum and inferoseptal regions, is strongly associated with suspected cardiac sarcoidosis and predicts death and major arrhythmic events [9,10]

FDG-PET provides complementary information by highlighting active inflammation. The joint SNMMI–ASNC consensus document outlines standardized protocols for using FDG-PET to detect cardiac sarcoid lesions and to monitor response to immunosuppressive therapy [11]. The 2024 AHA scientific statement emphasizes an integrated approach in which CMR identifies scar and structural involvement, while PET assesses inflammatory activity and helps guide the intensity and duration of immunosuppressive treatment [6].

In this case, the patient’s CMR findings alone were sufficient to classify him as having probable cardiac sarcoidosis, in line with these guidelines. FDG-PET may be pursued as an outpatient study to further characterize disease activity and guide potential steroid therapy.

### ICD therapy for secondary prevention

Patients with cardiac sarcoidosis and sustained VT are at high risk for recurrent arrhythmias and sudden death [5,7]. The 2017 AHA/ACC/HRS guideline provides a Class I recommendation for ICD implantation in patients with structural heart disease who have survived sustained VT or ventricular fibrillation not due to a reversible cause [5]. The HRS consensus specifically recommends ICD therapy for patients with cardiac sarcoidosis and spontaneous sustained VT, regardless of left ventricular ejection fraction [3,7].

Our patient clearly met these criteria: he had structural heart disease with mildly reduced LVEF, probable cardiac sarcoidosis on imaging, and recurrent episodes of sustained monomorphic VT. ICD implantation was therefore appropriate and consistent with current evidence-based recommendations.

## Conclusion

In patients presenting with sustained monomorphic VT, mildly reduced LVEF, minimal coronary artery disease, and a nonischemic scar pattern on CMR, cardiac sarcoidosis should be strongly considered. Contemporary guidelines acknowledge the low sensitivity and risk of endomyocardial biopsy and now permit a diagnosis of probable cardiac sarcoidosis based on clinical and imaging findings alone. CMR and FDG-PET have become central to this diagnostic strategy, and ICD implantation for secondary prevention is essential to reduce the risk of sudden cardiac death. This case highlights the importance of an imaging-guided, guideline-driven approach to unexplained ventricular arrhythmias.

## Author contributions

MS, FD, and FM contributed to writing the original draft. MS, FD, FM, DU, FZ, SMA, AA, YV, CTG, SL, AB, AH, and RN contributed to reviewing and editing the manuscript for important intellectual content and approval of the final manuscript.

## Declaration of generative AI and AI-assisted technologies in the manuscript preparation process

During the preparation of this work, the authors used ChatGPT in order to review literature and to rephrase text to improve grammar and clarity. After using this tool, the authors reviewed and edited the content as needed and take full responsibility for the content of the published article.

## Patient consent

Written informed consent for publication of their case was obtained from the patient.
